# Hidden Risks of Ultra-Processed Foods: How Health and Environmental Risk Perceptions Drive Sustainable Dietary Intentions in Taiwan

**DOI:** 10.3390/nu18101518

**Published:** 2026-05-10

**Authors:** Xiaozhong Cui, Yun-Chi Tsai, Tianmin Xu, Han-Shen Chen

**Affiliations:** 1 Department of Accounting, Jiaxing University, Jiaxing 314001, China; bellchung39@gmail.com (X.C.); 15157329035@163.com (T.X.); 2 Department of Health Industry Technology Management, Chung Shan Medical University, Taichung 40201, Taiwan; vickycai2527@gmail.com; 3 Department of Medical Management, Chung Shan Medical University Hospital, Taichung 40201, Taiwan

**Keywords:** ultra-processed food, dual-risk perception, theory of planned behavior, dietary behavior, front-of-pack labeling, sustainable diets

## Abstract

**Background/Objective:** Ultra-processed foods (UPFs) have become deeply embedded in global dietary patterns. However, their widespread consumption conceals the dual hidden risks of delayed physiological health effects and long-overlooked environmental externalities. Prior research has largely centered on health-driven dietary behaviors, with insufficient understanding of how perceptions of the environmental burden shape consumer choices, particularly in highly convenient, eating-out-dominated food environments. To address this gap, this study extends the theory of planned behavior (TPB) to examine how dual-risk perceptions influence intentions to reduce UPF consumption. **Methods:** Drawing on survey data from 362 Taiwanese consumers, this study analyzed the proposed theoretical model using structural equation modeling. **Results:** The findings show that (1) both health and environmental risk perceptions significantly and positively shape attitudes toward reducing UPF intake; (2) attitude, subjective norms, and perceived behavioral control (PBC) significantly increase reduction intentions, with subjective norms and attitude emerging as the strongest predictors; and (3) environmental awareness produces a counterintuitive diminishing marginal effect, negatively moderating the relationship between environmental burden perception and behavioral intention. **Conclusions:** These results extend the empirical foundation of the “green TPB” by demonstrating that the internalization of environmental costs complements traditional health motivations. The findings offer actionable implications for public health policy, including the implementation of front-of-pack warning labels and the use of the NOVA food classification system to advance sustainable diets.

## 1. Introduction

Ultra-processed foods (UPFs) have become firmly embedded in global dietary patterns due to their convenience, hyper-palatability, and low cost in recent years. In many high-income countries, UPFs account for more than half of the total daily energy intake [1]. A substantial body of epidemiological evidence links high UPF consumption to obesity, type-2 diabetes, cardiovascular diseases, and all-cause mortality [2]. In Taiwan’s highly convenient, eating-out-dominated food environment, characterized by a pervasive hand-shaken beverage culture and 24 h convenience stores, UPF intake among adolescents and young adults has risen sharply, alongside a decline in overall dietary quality [3,4].

Despite clear evidence of physiological harm, most dietary behavior research remains health-centric, leaving the environmental burden of UPFs comparatively underexamined. The global food supply chain accounts for approximately 26% of greenhouse gas emissions. UPFs impose additional environmental costs through energy-intensive processing, transnational cold-chain logistics, and extensive single-use plastic packaging [5,6]. Thus, the widespread consumption of UPFs conceals the dual hidden risks of delayed health harm and long-neglected environmental externalities. Because consumers typically prioritize price and convenience, a pronounced cognitive–behavioral gap persists regarding the environmental costs embedded in UPFs. This gap underscores the need for a robust theoretical model that explains how consumers balance immediate gratification with these dual risks.

UPF overconsumption extends beyond individual nutrition and reflects a broader behavioral challenge shaped by a low-cost, high-immediate-reward choice architecture. UPFs’ hyper-convenience, price advantages, and intense sensory appeal encourage short-term gratification while obscuring long-term physiological and environmental consequences. This dynamic exemplifies the well-documented attitude–behavior gap in dietary and sustainable consumption contexts [7,8,9]. Even when consumers recognize the dual hidden risks, perceived barriers (e.g., higher costs and limited access to healthier alternatives) often impede the translation of awareness into reduction behaviors.

The theory of planned behavior (TPB) offers a rigorous framework to analyze this cognitive–behavioral tension [10,11]. TPB posits that attitude, subjective norms, and perceived behavioral control (PBC) shape behavioral intentions. Although it is widely applied to health behaviors and green consumption, the TPB has rarely been used to explicitly conceptualize UPFs as a health–environment dual-risk commodity. The TPB enables the simultaneous evaluation of personal evaluations (attitudes), social influences (subjective norms), and perceived feasibility (PBC) in shaping dietary decisions within habituated and context-constrained environments.

The integration of the health risk perception and environmental burden perception into this framework is pivotal for both theoretical and practical reasons. Theoretically, while traditional dietary research primarily focuses on egoistic motivations (e.g., personal health benefits), UPFs represent a dual threat that links human physiology to global ecological stability. Exploring these two perceptions simultaneously allows for a more comprehensive understanding of the “egoistic–altruistic” tension in modern consumption, offering a nuanced perspective on the “dual-risk” framework. Practically, this approach provides policymakers with evidence-based insights to design multifaceted interventions. By examining whether environmental awareness can supplement health motivations, public health campaigns could leverage dual-themed messaging—emphasizing both personal well-being and environmental stewardship—to more effectively drive behavioral change toward minimally processed diets.

Building on this foundation, this study explores the integration of the health risk perception and environmental burden perception as key antecedents into the cognitive layer of the TPB framework. We examine how these dual-risk perceptions shape negative attitudes toward UPFs and influence reduction intentions through TPB pathways.

Accordingly, this study addresses the following three research questions:

- How do perceptions of delayed health hazards and environmental burdens influence attitudes toward reducing UPF consumption?
- How do attitude, subjective norms, and PBC jointly shape reduction intentions in a highly convenient food environment within an extended TPB framework?
- How do individual characteristics (e.g., environmental awareness and health involvement) and perceived control interact with these cognitive evaluations to moderate intention formation?

This study contributes to the literature by addressing these questions. Theoretically, it extends the literature on the green TPB by conceptualizing UPFs as a dual-risk commodity, thereby bridging dietary health psychology and sustainable consumption research to identify the complexities of consumer decision-making. The findings provide an empirical basis for discussing potential structural interventions, including front-of-pack warning labels, carbon footprint tracking, and the integration of the University of São Paulo’s NOVA food classification system into national dietary guidelines. These measures can support governments and industry actors in designing more effective, sustainable marketing strategies to counter entrenched UPF consumption patterns. To systematically categorize food products, this study utilizes the NOVA food classification system, which groups all foods into four categories based on the nature, extent, and purposes of industrial processing [1]. Group 4, which is specifically defined as ultra-processed foods (UPFs), consists of industrial formulations typically containing five or more ingredients, including additives such as flavor enhancers, colorants, and emulsifiers that are not commonly used in domestic kitchens [2]. In practice, this group encompasses widely consumed products such as carbonated sodas, mass-produced packaged snacks, reconstituted meat products (e.g., chicken nuggets and sausages), and instant powdered soups.

This classification is essential for assessing the multidimensional risks associated with modern diets; for instance, while high UPF exposure is a primary driver of adverse clinical health outcomes like obesity and cardiovascular diseases [12], the global food systems they characterize are also responsible for approximately 34% of total anthropogenic greenhouse gas emissions [13]. The novelty of this research lies in empirically demonstrating how these substantiated health and environmental risks interact within the Taiwanese context, where hyper-convenience often normalizes UPF consumption.

The findings provide an empirical basis for discussing potential structural interventions, including mandatory front-of-pack (FOP) warning labels, carbon footprint tracking, and the formal integration of the NOVA system into national dietary guidelines. These measures can support governments and industry actors in designing more effective, sustainable marketing strategies and public policies to counter entrenched and unsustainable UPF consumption patterns.

## 2. Literature Review and Hypothesis Development

### 2.1. Consumption Trends and Health Risks of UPFs

UPFs are defined as industrial formulations derived from food substances and additives and are typically characterized by a high energy density, hyper-palatability, and low nutritional value [1]. Rapid urbanization and the globalization of food retail have driven UPFs to dominate dietary patterns worldwide over the past three decades. In high-income countries, such as the United States, Canada, and the United Kingdom, UPFs account for 50–60% of daily energy intake, and middle-income countries are experiencing comparable growth [1]. In Taiwan, national nutrition surveys document a steady shift from traditional diets toward highly processed foods, particularly among adolescents and young adults [3].

To systematically analyze how these physiological risks translate into behavioral motivation, this study employs the theory of planned behavior (TPB) as its primary theoretical backbone, a framework that has been extensively validated for predicting health-related and sustainable food consumption [14]. Based on the logic of cognitive–affective consistency, individuals’ attitudes toward a behavior are shaped by their cognitive evaluation of the behavior’s consequences [15]. In the context of UPFs, as consumers internalize the delayed health harm, this risk perception serves as a critical cognitive antecedent that drives a negative evaluative response toward consumption. This integration aligns with the perspective that food choice is increasingly driven by a complex interplay between personal well-being and broader ethical evaluations.

A substantial body of epidemiological evidence links high UPF consumption to obesity, metabolic syndrome, type 2 diabetes, cardiovascular diseases, and all-cause mortality [2]. Randomized controlled trials further show that ad libitum UPF diets lead to excess caloric intake and rapid weight gain compared with minimally processed diets [16]. In this study, health risk perception is conceptually defined as an individual’s subjective assessment of these potential physiological harms and the long-term medical consequences associated with UPF consumption. As consumers increasingly recognize these delayed but serious health consequences, such risk perceptions are expected to generate more negative UPF evaluations. Despite the substantiated health risks, individuals frequently encounter a cognitive trade-off between long-term well-being and immediate utility. Empirical evidence suggests that functional attributes, particularly price and convenience, remain the most critical determinants of food choice for a majority of consumers, often overriding ethical or health concerns in retail environments [17,18]. Recent studies indicate that over 70% of consumers prioritize price over sustainable attributes when purchasing processed foods [19]. In this study, we argue that the “hidden” nature of metabolic risks is exacerbated by the hyper-convenience of UPFs, which effectively neutralizes the preventive impact of health risk perception. Thus, we formulated the following hypothesis:

**H1:**  *Consumers’ health risk perception of UPFs positively influences their attitude toward reducing UPF consumption*.

### 2.2. Environmental Burden of UPFs

Beyond physiological harm, UPF production and consumption impose substantial environmental costs. The global food supply chain accounts for approximately 26% of anthropogenic greenhouse gas emissions; within this system, UPFs intensify ecological pressures through energy-intensive processing, transnational cold-chain logistics, and extensive single-use plastic packaging [5]. In this context, transnational cold-chain logistics refers to the continuous, temperature-controlled supply chain required to distribute UPFs across international borders. This infrastructure is inherently energy-intensive and highly susceptible to temperature abuse during long-distance transportation and frequent loading/unloading, which significantly increases the carbon footprint and environmental burden associated with global food systems [13,20]. Shifting away from highly processed and resource-intensive foods can significantly reduce individual ecological footprints [6]. For example, lowering the intake of discretionary foods (largely UPFs) yields public health gains while reducing water depletion and carbon emissions [21]. These products are defined as foods and drinks not necessary to provide the nutrients required by the human body; they are typically energy-dense but nutrient-poor, such as sugar-sweetened beverages, confectionery, and salty snacks. According to Hadjikakou (2017) [21], discretionary foods, which largely overlap with ultra-processed foods (UPFs), account for a disproportionate share of diet-related environmental impacts, representing approximately 33% to 39% of the total life cycle water use, energy use, and greenhouse gas emissions.

Despite this evidence, consumer research has largely emphasized food categories (e.g., meat- vs. plant-based diets) rather than processing levels, leaving UPF environmental externalities comparatively underexplored [5]. In this study, environmental burden perception is conceptually defined as the extent to which an individual recognizes the ecological costs and negative environmental externalities, such as energy-intensive processing and excessive packaging, produced throughout the UPF supply chain. When consumers recognize the environmental burdens associated with UPFs, these perceptions should foster more favorable attitudes toward reducing consumption. In this study, UPFs are further conceptualized as carrying “dual hidden risks,” referring to the synergistic yet often obscured threats to both clinical health and ecological sustainability. These risks are considered “hidden” because they are secondary to the primary functional rewards, such as taste, convenience, and low cost, that dominate consumers’ immediate sensory experiences. As a result, individuals tend to overlook the long-term consequences associated with UPF consumption.

One critical dimension of these hidden risks lies in their environmental externalities. For instance, industrial food systems are responsible for approximately 34% of global greenhouse gas emissions [13]. However, such environmental impacts remain decoupled from the point of purchase due to discursive confusion and information asymmetry, which prevent consumers from translating awareness into meaningful action [22]. Consequently, consumers often fail to associate their food choices with broader ecological impacts. Recognizing these hidden environmental costs is therefore a crucial psychological prerequisite for fostering sustainable dietary intentions. Therefore, we propose the following hypothesis:

**H2:**  *Consumers’ perception of the environmental burden of UPFs positively influences their attitude toward reducing UPF consumption*.

### 2.3. The TPB: Bridging the Cognitive–Behavioral Gap

The TPB offers a rigorous framework to systematically explain how dual-risk perceptions translate into dietary behavioral change. Proposed by Ajzen (1991) [10], the TPB posits that attitude, subjective norms, and PBC shape behavioral intentions. Although the TPB is widely applied in sustainable food research [8,12], food consumption is highly habitual and embedded in contemporary food environments. The framework enables the simultaneous examination of personal evaluations, social influences, and PBCs.

Applying this framework to the current research, the TPB is employed to explain the psychological mechanisms underlying the intention to reduce UPF consumption. Specifically, attitude refers to the negative evaluation of UPFs as dual-risk commodities; subjective norms capture the perceived social pressure to adopt healthier, less-processed diets within Taiwan’s eating-out culture, where social circles heavily influence food choice [23]. Finally, PBC represents the individual’s perceived ability to overcome structural barriers, such as the high density of convenience stores and the ubiquity of affordable UPF alternatives [24]. This model is extended by integrating risk perceptions as distal antecedents that shape these core TPB constructs, thereby providing a context-sensitive explanation for dietary decision-making [25].

#### 2.3.1. Attitude and Its Mediating Role

Cognitive beliefs produce evaluative attitudes within the TPB, which in turn shape behavioral intentions [26]. When consumers recognize the environmental externalities of UPFs, this cognitive appraisal is expected to foster more negative attitudes toward consumption, functioning as a key psychological mechanism. Empirical research indicates that environmental concern increases the intention to adopt sustainable alternatives (e.g., plant-based meats) through attitudinal mediation [27]. Accordingly, attitude should operate as both a direct predictor of intention and a mediator linking environmental burden perception to reduction intention. In this research, attitude is conceptually defined as the degree to which an individual forms a favorable or unfavorable evaluation or appraisal of reducing their UPF intake. Therefore, we propose the following hypotheses:

**H2a:**  *Attitude mediates the relationship between environmental burden perception and the intention to reduce UPF consumption*.

**H3:**  *Consumers’ attitudes toward reducing UPFs positively influence their behavioral intentions*.

#### 2.3.2. Role of Subjective Norms

Dietary behavior is socially embedded. In highly convenient food environments where UPF consumption functions as a default norm, individuals may encounter social resistance when seeking to reduce intake. Subjective norms (i.e., perceived expectations or support from significant others, peers, family, nutritionists, or environmental advocates) can counteract entrenched habits. When sustainable and healthy diets receive strong social endorsement, this support strengthens the motivation to change consumption patterns [28]. In the context of this study, subjective norms are conceptually defined as the perceived social pressure from significant others, such as family, friends, and peers, to perform or refrain from the reduction in UPF consumption. As individuals perceive that their social circle values dietary health and environmental stewardship, they are more likely to align their intentions with these social expectations. Thus, we propose the following hypothesis:

**H4:**  *Subjective norms positively influence consumers’ intention to reduce UPF consumption*.

#### 2.3.3. PBC and Moderation

PBC reflects perceived self-efficacy and access to the resources necessary to perform a behavior. Meta-analytical evidence identifies PBC as a strong predictor of health-related intentions [29]. Beyond its direct effect, PBC may moderate the well-documented “attitude–behavior gap” in environmental contexts [7,9]. Even when consumers recognize the environmental risks of UPFs, limited affordability, a limited availability of alternatives, or time constraints may impede intention formation. In this study, perceived behavioral control (PBC) is conceptually defined as an individual’s perception of the ease or difficulty of reducing UPF consumption, reflecting both internal self-efficacy and external facilitating or inhibiting conditions. Conversely, higher PBC increases the likelihood that the environmental burden perception translates into reduction intention. Accordingly, we propose the following hypotheses:

**H5:**  *PBC positively influences consumers’ intention to reduce UPF consumption*.

**H5a:**  *PBC positively moderates the relationship between environmental burden perception and behavioral intention; specifically, the effect is stronger when PBC is high*.

### 2.4. Sociodemographic Determinants of UPF Consumption

UPF consumption is not homogeneous across populations; it varies systematically according to sociodemographic characteristics. A recent systematic review of 32 countries showed that younger individuals, urban residents, and unmarried adults reported higher UPF intake [4]. These patterns suggest that younger and highly urbanized groups are more structurally embedded in convenience-oriented, ready-to-eat food environments [30]. Empirical evidence from Taiwan similarly indicates that higher levels of urbanization are associated with more frequent UPF consumption [3]. Furthermore, sociodemographic factors have been proven to be the most powerful predictors of consumer behavior, even surpassing the influences of lifestyle and individual knowledge level [31]. Accordingly, sociodemographic factors should be incorporated into the behavioral model to clarify their role in influencing reduction intentions. We propose the following hypotheses:

**H6a:**  *Age significantly influences consumers’ intentions to reduce UPF consumption*.

**H6b:**  *Education level significantly influences consumers’ intentions to reduce UPF consumption*.

**H6c:**  *Monthly personal income significantly influences consumers’ intentions to reduce UPF consumption*.

### 2.5. Roles of Personal Traits: Health Involvement and Environmental Awareness

Beyond sociodemographic influences, psychological traits shape how consumers interpret risk information and form UPF attitudes. In consumer research, “involvement” refers to the degree of personal relevance of a specific issue and cognitive engagement [32].

Health involvement reflects sustained attention to dietary well-being and is defined as the degree of personal relevance an individual assigns to their health. Individuals with high health involvement are more sensitive to food-related risks and are more likely to associate UPFs with adverse physiological outcomes, including obesity and metabolic syndrome [2,33]. In contrast, environmental awareness captures general sensitivity to ecological protection. Consumers with greater environmental awareness are more likely to recognize the carbon footprint and plastic waste associated with UPF production and packaging [5]. Therefore, the following traits function as key antecedents to risk perception:

**H7:**  *Environmental awareness positively influences consumers’ perception of the environmental burden of UPFs*.

**H8:**  *Health involvement positively influences consumers’ perception of the health risks of UPFs*.

Environmental awareness may also act as a moderator in the process of intention formation. Although a higher environmental burden perception typically increases reduction intention, the strength of this effect may vary according to an individual’s baseline environmental awareness. Examining this interaction elucidates the boundary conditions of sustainable dietary interventions. Therefore, we propose the following hypothesis:

**H9:**  *Environmental awareness significantly moderates the relationship between the environmental burden perception and the intention to reduce UPF consumption*.

### 2.6. Research Framework

**Figure 1:**
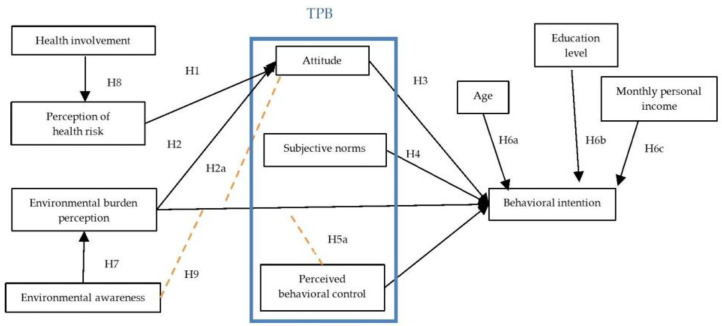
Conceptual model.

This study proposes an integrated conceptual model that combines the extended TPB, dual-risk perceptions (health and environmental risks), and personal traits (health involvement and environmental awareness) based on the literature and theoretical reasoning. The model is designed to systematically explain consumers’ intentions to reduce UPF consumption. Figure 1 illustrates the hypothesized relationships and the moderating role of environmental awareness.

## 3. Materials and Methods

### 3.1. Participants and Procedure

This study employed a cross-sectional, quantitative design. The data were collected through an online survey administered from 26 October to 9 November 2025. Specific inclusion criteria were applied to ensure the relevance and quality of the data: participants were required to be at least 18 years of age and currently residing in Taiwan to ensure familiarity with the local food retail environment and its high density of convenience stores. Convenience sampling was conducted through major social media and messaging platforms (e.g., Facebook, Instagram, and LINE) to reach Taiwanese consumers with prior experience purchasing and consuming UPFs. Participation was entirely voluntary, and no incentives were provided to ensure that the responses reflected genuine consumer attitudes rather than reward-motivated behavior. The requirement for ethical review and approval was waived for this study in accordance with the regulations of the National Science and Technology Council (NSTC) in Taiwan, as it was a non-interventional, anonymous survey that did not involve vulnerable populations. Before participation, the respondents reviewed an informed consent statement outlining the purpose of this study, anonymity protections, and their right to withdraw at any time. A total of 421 responses were obtained. Due to the nature of open-link distribution across social platforms, an exact response rate could not be determined. However, to handle potential inattentive or careless responding, a rigorous manual screening process was conducted to identify “straight-lining” patterns, where respondents provided identical ratings across all items. After removing invalid cases, 362 valid responses were retained for the analysis. The minimum sample size was determined based on the respondent-to-item ratio recommended for structural equation modeling (SEM). According to Hair et al. (2010) and Kline (2015) [34,35], a sample-to-item ratio of at least 10:1 is an appropriate target to ensure stable parameter estimates. Given that our measurement model contains 25 items, the retained sample of 362 significantly exceeds the required threshold, providing sufficient statistical power for the structural analysis.

### 3.2. Measures

The questionnaire was developed from scales validated in prior research and adapted to the UPF context. All items were rated on a 7-point Likert scale ranging from 1 (“strongly disagree”) to 7 (“strongly agree”). Given its focus on the emerging dual-risk perceptions in a unique retail environment, this research can be considered a perspective study that provides a baseline for future cross-cultural comparisons. The instrument included the following constructs:

- Health Risk Perception (3 items) and Health Involvement (3 items): Adapted from Adasme-Berríos et al. [36] and Jakubowska et al. [37] to assess the perceived physiological harm of UPFs and the personal relevance of dietary health.
- Environmental Burden Perception (3 items) and Environmental Awareness (3 items): Adapted from Ilieva et al. [38], Alam et al. [8], Carfora et al. [39], and Chen [27] to measure the recognition of the ecological impacts and general environmental sensitivity of UPFs.
- TPB Constructs—Attitude (3 items), Subjective Norms (3 items), and PBC (3 items): Focuses on reducing UPF consumption; adapted from Alam et al. [8], Ilieva et al. [38], Chang et al. [40], and Jakubowska et al. [37].
- Behavioral Intention (4 items) measures the intention to reduce UPF intake, and was adapted from Carfora et al. [39], Chen [27], and Ilieva et al. [38].

### 3.3. Statistical Analysis

The data were analyzed using IBM SPSS Statistics v. 27 and AMOS 31 software (IBM Corp., Armonk, NY, USA). Descriptive statistics summarize the sociodemographic profile of the sample. Reliability and convergent validity were assessed using Cronbach’s α, composite reliability (CR), and average variance extracted (AVE) to evaluate measurement consistency. Structural equation modeling (SEM) with the maximum likelihood (ML) estimation was then applied to assess the overall model fit and test the hypothesized structural paths and moderating effects within the extended TPB framework. SEM enables the simultaneous estimation of multiple interrelated relationships while accounting for measurement error.

## 4. Results

### 4.1. Sample Characteristics

A total of 421 responses were collected, of which 362 were retained after data screening. The sample was evenly distributed by gender (51.1% male; 48.9% female). Most respondents were aged 18–54 years, with the 18–24 age group representing the largest segment (27.1%). Regarding education, 62.4% held a university degree. Table 1 presents the detailed sociodemographic characteristics.

### 4.2. Measurement Model Evaluation

To ensure the integrity of the data, Harman’s single-factor test was performed prior to evaluating the measurement model to assess potential common method variance (CMV). The results indicated that the first factor explained 47.01% of the total variance. As this value falls below the recommended 50% threshold [41,42,43], it suggests that the results are not significantly affected by common method bias. Internal consistency, convergent, and discriminant validity were evaluated to assess the reliability and validity of the measurement model (Table 2). The standardized factor loadings ranged from 0.714 to 0.841, exceeding the recommended threshold of 0.50. Cronbach’s α and CR values for all constructs exceeded 0.70, indicating strong internal consistency. In addition, AVE values exceeded 0.50 for all constructs, indicating adequate convergent validity [44,45,46].

The discriminant validity was evaluated using the Fornell–Larcker criteria. The square root of the AVE for each construct (bolded on the diagonal) exceeded its correlations with all other constructs, indicating adequate discriminant validity (Table 3).

### 4.3. Structural Model and Fit Indices

To test the hypothesized relationships, the structural equation model was estimated using ML. The model fit indices indicated an acceptable fit: chi-square to degrees of freedom ratio (χ^2^/df) = 2.283, comparative fit index (CFI) = 0.944, incremental fit index (IFI) = 0.944, normed-fit index (NFI) = 0.905, standardized root mean square residual (SRMR) = 0.0395, and root mean square error of approximation (RMSEA) = 0.060. Although the goodness-of-fit index (GFI) (0.876) and adjusted goodness-of-fit index (AGFI) (0.839) fell slightly below the conventional 0.90 threshold, these values are considered acceptable for complex models with larger samples when the incremental fit indices (e.g., CFI and NFI) are strong [47,48].

### 4.4. Hypothesis Testing (Main Effects)

To rigorously evaluate the proposed conceptual framework, a structural equation modeling (SEM) approach was employed using AMOS 26.0. This method was selected for its capacity to simultaneously examine multiple interrelated dependence relationships while accounting for measurement errors. Before hypothesis testing, a two-step modeling approach was followed: the measurement model was first validated through a Confirmatory Factor Analysis (CFA) to ensure construct validity, followed by the evaluation of the structural model using maximum likelihood estimation.

**Figure 2:**
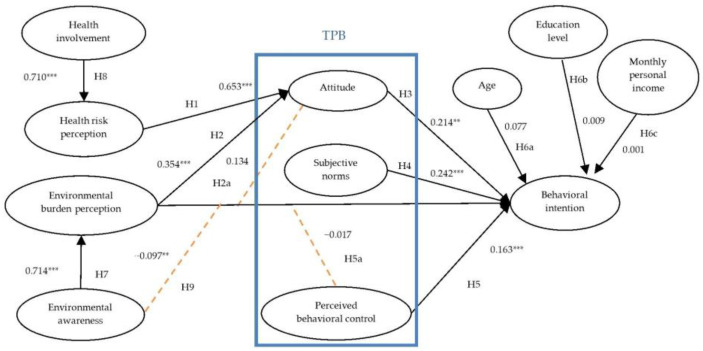
Results of the analysis of the structural model. Path coefficients are presented as standardized estimates (*β*). *** *p* < 0.001 and ** *p* < 0.05.

The results of the path analysis are reported in Table 4 and Figure 2. The structural model provides strong evidence for the proposed dual-risk mechanism. Environmental awareness positively predicted environmental burden perception (*β* = 0.714, *p* < 0.001), supporting **H7**. Health involvement similarly predicted health risk perception (*β* = 0.710, *p* < 0.001), supporting **H8**. Health risk perception (*β* = 0.653, *p* < 0.001) and environmental burden perception (*β* = 0.354, *p* < 0.001) positively influenced attitudes toward reducing UPF consumption, supporting **H1** and **H2**. The notably stronger effect of health risk perception indicates that personal physiological well-being remains the primary driver of evaluative appraisal, although ecological concerns are increasingly being integrated into the consumer’s cognitive layer.

Within the TPB framework, attitude (*β* = 0.214, *p* = 0.008), subjective norms (*β* = 0.242, *p* < 0.001), and PBC (*β* = 0.163, *p* = 0.005) exerted significant positive effects on behavioral intention, supporting **H3**, **H4**, and **H5**. Notably, subjective norms emerged as the strongest predictor of intention, reflecting the powerful role of social pressure and collective dietary expectations in Taiwan’s eating-out culture. Regarding the role of sociodemographic characteristics (**H6a**–**H6c**), each factor was modeled as an individual observed variable to examine its distinct influence on behavioral intention. The results indicated that age (*β* = 0.077, *p* = 0.139), education level (*β* = 0.009, *p* = 0.818), and monthly personal income (*β* = 0.001, *p* = 0.977) did not significantly predict the intention to reduce UPF consumption. Consequently, **H6a**, **H6b**, and **H6c** were not supported.

### 4.5. Mediating and Moderating Effects

**Figure 3:**
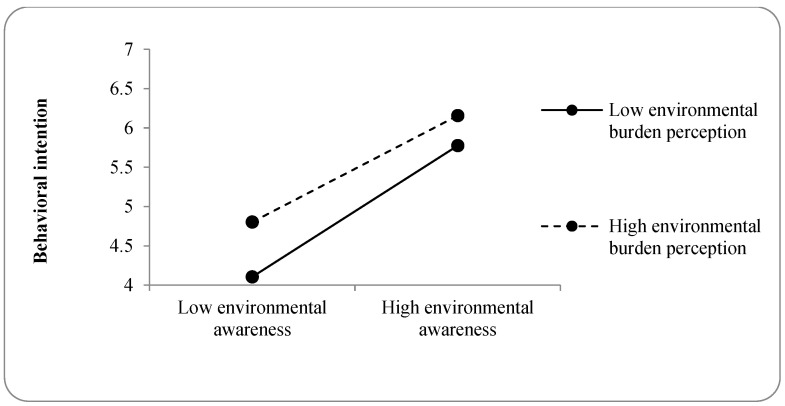
Effect of environmental awareness on the relationship between environmental burden perception and behavioral intention.

Mediation (**H2a**) and moderation (**H5a** and **H9**) analyses were conducted to examine indirect effects and boundary conditions. The mediation results indicate that the indirect effect of environmental burden perception on behavioral intention through attitude was not statistically significant (indirect effect = 0.298, 95% CI [−0.038, 0.834]), as the confidence interval included zero (Table 5). Accordingly, **H2a** was not supported. The moderating effect of PBC (**H5a**) on the relationship between environmental burden perception and behavioral intention was also nonsignificant (*β* = −0.017, *p* = 0.734). In contrast, environmental awareness moderated this relationship (*β* = −0.097, *p* = 0.012), supporting **H9**. A simple slope analysis (Figure 3) indicates a diminishing marginal effect: the positive association between environmental burden perception and intention is weaker among individuals with high environmental awareness, whereas increases in environmental burden perception are associated with a steeper increase in the intention to reduce UPF consumption for those with lower environmental awareness.

## 5. Discussion

### 5.1. Dual Risks: Health and Environmental Perceptions

The findings indicate that perceptions of both the health risk and environmental burden significantly shape negative attitudes toward UPF consumption, supporting **H1** and **H2**. Consistent with prior clinical and epidemiological research [2,10], health risk perception exerts a stronger influence, likely due to its direct personal relevance and perceived immediacy [49]. This observation aligns with the conclusions reported by Sánchez-Bravo et al. (2020) [18], who noted that while sustainability concerns are increasingly prevalent, personal health attributes remain the primary determinant in food choice categories. Furthermore, the dual-risk mechanism shown here is supported by evidence identifying that biospheric and egoistic values concurrently drive attitudes toward sustainable food [27]. Our results demonstrate that consumers are beginning to categorize UPFs as a “complex risk” where dietary choices are viewed as a means to mitigate broader ecological threats, a shift also evidenced in the recent analysis of food processing perceptions [19]. Importantly, the environmental burden perception also influences food evaluation. Consumers who recognize the carbon footprint and plastic waste associated with UPFs assign lower sustainability value to these products, consistent with pro-environmental attitude formation theories [23,50]. In addition, personal traits—health involvement and environmental awareness—significantly enhance these dual-risk perceptions (**H7** and **H8**), aligning with involvement theory [32,33].

### 5.2. Decoding TPB Pathways and the Attitude–Intention Gap

Within the TPB framework, attitude, subjective norms, and PBC significantly increase the intention to reduce UPF consumption (**H3**, **H4**, and **H5**). However, the mediating role of attitude between environmental burden perception and intention (**H2a**) was not supported. This finding highlights an attitude–intention gap: although environmental information strengthens negative UPF evaluations, attitude alone does not translate into behavioral intention without structural reinforcement. This finding resonates with the classic framework, which suggests that low perceived availability often hinders the translation of positive attitudes into actual purchase intentions [51]. The subjective norm emerged as a particularly strong predictor. UPF consumption is socially normalized in highly convenient, eating-out-dominated contexts, such as Taiwan, and support from peers, nutritionists, or environmental advocates becomes critical for behavioral change [39]. The predictive power of subjective norms in our model corroborates the finding that in collective dining cultures, social trust and normative pressure are pivotal in dietary transitions [15]. Similarly, PBC facilitates intention formation by mitigating structural barriers, including the cost and availability of whole-food alternatives [52]. Nevertheless, PBC did not moderate the relationship between environmental burden perception and intention (**H5a**). This lack of moderation suggests that situational factors may override perceived control in commercialized food environments [53]. In such a commercialized food environment, the translation of environmental concern into action remains constant regardless of an individual’s perceived level of control, underscoring the dominance of external barriers over personal agency.

Our results align with findings from other Asian contexts; for instance, Jia and Liu (2025) and Sajjad et al. (2023) [19,54] also identified that subjective norms and health consciousness are pivotal in driving intentions toward functional and fast foods in China and Pakistan, respectively. Interestingly, while Nystrand and Olsen (2020) [24] showed that PBC had a limited or even negative impact on functional food intentions in Norway, our study in Taiwan similarly observed that external structural barriers, such as a high convenience store density, can neutralize the effect of individual agency. This result suggests that the “environmental dominance” over dietary intention is a cross-cultural phenomenon observed in both Western and Eastern food systems.

### 5.3. Boundary Condition: Diminishing Marginal Effect of Environmental Awareness

The moderating role of environmental awareness (**H9**) is a key finding. The interaction indicates a diminishing marginal effect: additional environmental burden information produces smaller gains in reduction intention among consumers with a high baseline environmental awareness. In contrast, consumers with a lower environmental awareness display a stronger positive response to a perceived increase in the environmental burden. This pattern underscores the heterogeneity of information processing across consumer segments. Importantly, the methodological robustness of this interaction is supported by multicollinearity diagnostics, which yielded a Variance Inflation Factor (VIF) of 1.000 and a tolerance of 1.000. These ideal values, combined with the use of mean-centering for the interaction components, confirm that the observed diminishing marginal effect is a substantive psychological phenomenon rather than a statistical artifact or a result of multicollinearity. Finally, **H6a**, **H6b**, and **H6c** did not significantly predict the reduction intention, reinforcing the view that UPF consumption represents a structural challenge across demographic groups [1].

### 5.4. Theoretical Contributions

This study offers two primary theoretical contributions. First, health psychology and sustainable consumption are connected by conceptualizing UPFs as a dual-risk commodity. Although prior dietary studies emphasize physiological outcomes, this model incorporates environmental externalities into the cognitive layer of the TPB, extending the “green TPB” framework to food processing levels. Second, this study advances the understanding of the attitude–intention gap. The findings propose a more context-sensitive behavioral model for commercialized food environments by identifying the roles of subjective norms and environmental awareness as a boundary condition (i.e., diminishing marginal effect).

### 5.5. Practical and Policy Implications

The results support a shift from individual-level education to structural intervention. Because PBC and subjective norms significantly influence reduction intentions, risk awareness alone is insufficient. Policymakers should reduce the structural barriers to dietary change. The recommended actions include the following:

- Implementing mandatory front-of-pack (FOP) warning labels and incorporating the NOVA classification system into national dietary guidelines to simplify UPF identification.
- Subsidizing minimally processed foods and taxing resource-intensive UPFs to strengthen PBCs.
- Mobilizing social influence by engaging environmental advocates and nutrition professionals to normalize reduced UPF consumption.

## 6. Conclusions and Implications

### 6.1. Conclusions

This study examined the psychological mechanisms underlying consumers’ intentions to reduce UPF consumption in a highly convenient food environment. By integrating the dual hidden risks of health hazards and environmental burdens into the extended TPB, the study shows that both risk perceptions significantly shape negative attitudes toward UPFs. However, attitude alone does not necessarily translate into reduction intention; structural support is required. Subjective norms emerged as the strongest predictor of intention, providing the social reinforcement necessary to counter entrenched dietary habits, while attitude serves as a critical evaluative driver. The diminishing marginal effect of environmental awareness further indicates heterogeneity in how consumer segments process sustainability information.

### 6.2. Limitations and Future Research

Several limitations warrant attention. First, the cross-sectional, self-report design captures intention rather than actual consumption behavior. Future research should employ longitudinal designs or objective purchasing data to address the intention–behavior gap. Second, although this study strictly adhered to ethical principles regarding anonymity and informed consent, the requirement for ethical review and approval was waived in accordance with local regulations for non-interventional surveys; however, future studies involving more sensitive data may benefit from formal institutional oversight. Third, the geographic focus on Taiwan’s unique retail structure—characterized by a high density of convenience stores—may limit the generalizability of these findings to regions with different food distribution systems. Nevertheless, this focus provides a critical perspective on “convenient” environments, offering a baseline for potential extension to other East Asian contexts or urban centers with similar infrastructures. Furthermore, convenience sampling may limit generalizability; cross-cultural studies could clarify how varying social norms influence UPF consumption. Fourth, the reliance on self-reported data means that risk perceptions and dietary intentions may be subject to social desirability bias, where participants might over-report virtuous intentions. Finally, future research may use CEs to examine how consumers make trade-offs between price, convenience, FOP labels, and environmental footprint when choosing between UPFs and minimally processed alternatives.

## Figures and Tables

**Table 1:** Sociodemographic characteristics of the participants.

N = 362	Characteristics	Frequency (*n*)	Percentage (%)
Gender	Male	185	51.1%
	Female	177	48.9%
Age (years)	Under 18	1	0.3%
	18–24	98	27.1%
	25–34	68	18.8%
	35–44	83	22.9%
	45–54	82	22.7%
	55–64	24	6.6%
	65 and above	6	1.7%
Education level	Junior high school or below	5	1.4%
	High school/Vocational school	66	18.2%
	College/University	226	62.4%
	Master’s degree or higher	65	18.0%
Monthly personal income (NT$)	Under 20,000	96	26.5%
	20,001–40,000	66	18.2%
	40,001–60,000	98	27.1%
	60,001–80,000	54	14.9%
	Above 80,000	48	13.3%

Note: NT$ = New Taiwan dollars.

**Table 2:** Reliability and validity of the proposed measurement model.

Constructs	Items	Standardized Factor Loading	CR	AVE	Cronbach’s α
Health risk perception	1. I believe that consuming ultra-processed foods may be harmful to my health.	0.758	0.822	0.606	0.815
	2. I worry that ultra-processed foods are not safe and reliable.	0.816			
	3. I am concerned that consuming ultra-processed foods may not be beneficial to me.	0.760			
Environmental burden perception	1. I believe that the production of ultra-processed foods causes more environmental pollution.	0.788	0.804	0.578	0.799
	2. When purchasing ultra-processed foods, I consider how my consumption might affect the environment and communities.	0.747			
	3. Reducing the consumption of ultra-processed foods improves the environment.	0.746			
Attitude	1. I believe that reducing the consumption of ultra-processed foods is beneficial to my health.	0.756	0.796	0.566	0.795
	2. I believe that purchasing ultra-processed foods is an unwise choice.	0.770			
	3. I believe that the quality of ultra-processed foods is inferior to that of other foods.	0.730			
Subjective norms	1. Professional opinions (e.g., nutritionists) influence my willingness to buy ultra-processed foods.	0.832	0.808	0.585	0.805
	2. Opinions from individuals or peers influence my desire to purchase ultra-processed foods.	0.727			
	3. Appeals from environmental organizations have influenced my willingness to buy ultra-processed foods.	0.730			
Perceived behavioral control	1. I know how to select products to avoid ultra-processed foods.	0.801	0.791	0.558	0.791
	2. I pay attention to the ultra-processed food prices.	0.714			
	3. I have the opportunity to purchase non-UPF foods	0.723			
Health involvement	1. To maintain my health, I carefully choose my food.	0.839	0.863	0.678	0.863
	2. I consider myself very health-conscious.	0.805			
	3. I frequently consider health-related issues when eating.	0.825			
Environmental awareness	1. I usually pay special attention to environmental protection issues.	0.807	0.868	0.687	0.868
	2. I choose environmentally friendly foods.	0.839			
	3. When shopping, I pay attention to whether a product is eco-friendly.	0.841			
Behavioral intention	1. I avoid buying ultra-processed foods to reduce packaging’s environmental burden.	0.794	0.868	0.623	0.869
	2. When product quality is comparable, I prefer natural foods over ultra-processed foods.	0.759			
	3. I would buy natural foods even if they are more expensive than those processed in ultra-processed foods.	0.768			
	4. I intend to reduce the purchase of ultra-processed foods within the next month.	0.833			

Notes: CR = composite reliability; AVE = extracted average variance. All standardized factor loadings are significant at *p* < 0.001.

**Table 3:** Correlation matrix and square roots of the AVE of the measurement model.

Constructs	Mean	SD	1	2	3	4	5	6	7	8
1. Health risk perception	5.247	1.131	**0.778**							
2. Environmental burden perception	5.070	1.031	0.700	**0.761**						
3. Attitude	5.025	1.005	0.731	0.750	**0.752**					
4. Subjective norms	4.994	1.172	0.575	0.603	0.637	**0.765**				
5. Perceived behavioral control	5.106	1.004	0.600	0.598	0.616	0.613	**0.747**			
6. Health involvement	5.057	1.255	0.537	0.545	0.584	0.591	0.642	**0.823**		
7. Environmental awareness	4.724	1.171	0.522	0.582	0.568	0.612	0.601	0.702	**0.829**	
8. Behavioral intention	5.164	1.156	0.629	0.614	0.640	0.657	0.661	0.743	0.789	**0.789**

Note: SD = standard deviation. Bold values on the diagonal represent the square roots of the average variance extracted (AVE); off-diagonal values represent the Pearson correlation coefficients between constructs.

**Table 4:** Path analysis and hypothesis-testing results.

Hypothesis/Path	B	S.E.	C.R.	*p*-Value	*β*	Result
**H1:** Health risk perception → Attitude	0.480	0.075	6.370	<0.001	0.653	Supported
**H2:** Environmental burden perception → Attitude	0.251	0.064	3.938	<0.001	0.354	Supported
**H3:** Attitude → Behavioral intention	0.224	0.085	2.639	0.008	0.214	Supported
**Direct effect:** Environmental burden perception → Behavioral intention	0.063	0.049	1.292	0.692	0.134	Not supported
**H4:** Subjective norms → Behavioral intention	0.913	0.240	3.808	<0.001	0.242	Supported
**H5:** Perceived behavioral control → Behavioral intention	0.613	0.217	2.824	0.005	0.163	Supported
**H5a:** Environmental burden perception × Perceived behavioral control → Behavioral intention	−0.017	0.050	−0.340	0.734	−0.017	Not supported
**H6a:** Age → Behavioral intention	0.044	0.030	1.479	0.139	0.077	Not supported
**H6b:** Education level → Behavioral intention	0.011	0.050	0.230	0.818	0.009	Not supported
**H6c:** Monthly personal income → Behavioral intention	0.001	0.028	0.029	0.977	0.001	Not supported
**H7**: Environmental awareness → Environmental burden perception (EBP)	3.622	0.297	12.183	<0.001	0.714	Supported
**H8:** Health involvement → Health risk perception	3.484	0.300	11.634	<0.001	0.710	Supported
**H9:** Environmental awareness × Environmental burden perception → Behavioral intention	−0.093	0.037	−2.505	0.012	−0.097	Supported

Notes: B = unstandardized coefficient; S.E. = standard error; C.R. = critical ratio; *β* = standardized coefficient.

**Table 5:** Summary of the mediating effects.

Paths	Estimate	*p*-Value (BC/PC)	95% CI (BC)	95% CI (PC)
**Indirect effect**				
Environmental burden perception → Attitude → Behavioral intention	0.298	0.093/0.119	[−0.038, 0.834]	[−0.053, 0.785]
**Direct effect**				
Environmental burden perception → Attitude	0.697	0.001/0.001	[0.506, 0.862]	[0.500, 0.857]
Environmental burden perception → Behavioral intention	0.134	0.692/0.698	[−0.565, 0.790]	[−0.568, 0.789]
Attitude → Behavioral intention	0.428	0.113/0.199	[−0.079, 0.966]	[−0.081, 0.961]
**Total effect**				
Environmental burden perception → Behavioral intention	0.432	0.002/0.002	[0.143, 0.764]	[0.140, 0.758]

Notes: BC = bias-corrected percentile method; PC = percentage method; CI = confidence interval.

## Data Availability

The original contributions presented in this study are included in the article; further inquiries can be directed to the corresponding author.

## Abbreviations

AVE	Average variance extracted
CR	Composite reliability
ML	Maximum likelihood
PBC	Perceived behavioral control
SEM	Structural equation modeling
TPB	Theory of planned behavior
UPF	Ultra-processed foods
